# Clinical radiation audits as a tool for the optimization of radiation exposure in cardiac electrophysiology procedures

**DOI:** 10.1016/j.zemedi.2025.04.003

**Published:** 2025-04-25

**Authors:** Lorraine Sazgary, Eleni Theano Samara, Anja Stüssi, Natalia Saltybaeva, Matthias Guckenberger, F. Ruschitzka, Thomas Wolber, Nadine Molitor, Fu Guan, Gonca Suna, Julia Hermes-Laufer, Alexander Breitenstein, Corinna B. Brunckhorst, Firat Duru, Ardan M. Saguner

**Affiliations:** a Department of Cardiology, University Hospital Zurich, University of Zurich, Zurich, Switzerland; b Radiation Protection Unit, University Hospital Zurich, University of Zurich, Zurich, Switzerland; c Department of Radiation Oncology, University Hospital Zurich, University of Zurich, Zurich, Switzerland; d Center for Translational and Experimental Cardiology (CTEC), University of Zurich, Zurich, Switzerland; e Center for Integrative Human Physiology, University of Zurich, Zurich, Switzerland

**Keywords:** radiation, audit, electrophysiology, cardiology

## Abstract

**Background:**

Clinical radiation audits are useful to reduce ionizing radiation in clinical practice. The first Swiss radiation audit in Cardiology took place at the University Heart Center Zurich in 2019.

**Objectives:**

To compare local diagnostic reference levels (DRLs) in cardiac electrophysiology (EP) procedures to the currently available national DRLs and to examine patient radiation exposure before and after the clinical radiation audit.

**Methods:**

Retrospective study including 775 patients undergoing EP procedures from 2018- 2020. Main recommendations of the clinical audit were the regular use of collimation, semitransparent filters, the reduction of cine images and frame rates. Patient radiation exposure was evaluated with cumulative doses, fluoroscopy times and dose-area product (DAP). Secondary endpoints were acute procedural success rates and 30-day complications.

**Results:**

447 (57.5%) patients prior to, and 328 (42.3%) after the audit were included. Cryoballoon pulmonary vein isolation (PVI) was performed in 14.1%, PVI radiofrequency (RF) ablation in 26.8%, RF ablation of right-sided supraventricular tachycardia in 32.1%, other procedures in 27% of cases. Local DRLs for the DAP were below national DRLs (1 Gy cm^2^ vs 150 Gy cm^2^ for AVNRT/AVRT ablation). After the audit, there was a significant radiation reduction for right-sided supraventricular tachycardia ablation (cumulative dose: 4.8 mGy vs 2.1 mGy and fluoroscopy times: 210 seconds vs 107 seconds, p < 0.001) and PVI with RF (50.4 mGy vs 29.5 mGy, and 378 seconds vs 191 seconds, p < 0.003; respectively). No significant differences were found in acute procedural success rates or 30-day complications.

**Conclusions:**

The clinical radiation audit was associated with a significant reduction of patient radiation exposure for right-sided supraventricular tachycardia ablation and PVI with RF.

## Introduction

Through the use of fluoroscopy and nuclear imaging cardiologists are responsible for up to 43% of the total radiation exposure experienced by the average US citizen [1]. Considering that fluoroscopy is the primary imaging technique in interventional cardiology and approximately 300.000 electrophysiology procedures are performed annually in Europe [2], it is essential to keep radiation exposure as low as reasonably achievable [3,4].

The hazards of ionizing radiation include stochastic effects, such as cancer risk, and deterministic effects, such as skin injuries and cataracts [4,5]. Often, multiple procedures are performed per patient and complex anatomical situations may require more fluoroscopy, which contributes to the cumulative risk for both patients and staff making dose optimization essential [1]. Therefore, various measures are implemented in the interventionalists’ everyday life to reduce radiation exposure ranging from personal shielding by wearing at least a lead apron and a thyroid collar, to using protective shields, and technical strategies like low-dose fluoroscopy, 3D-mapping systems and intracardiac ultrasound (ICE) [3,6,7,8].

As a new development in Switzerland and mandated by Swiss law, clinical radiation audits are conducted in cardiology departments as a quality management tool to ensure the optimal use of ionizing radiation in clinical practice since 2019 [9]. During these audits, a team of external peer auditors systematically assesses radiation practices to optimize processes and resources. Diagnostic reference levels (DRLs) are used as a benchmark to ensure that the amount of ionizing radiation used for procedures remains within acceptable levels. If patient dose values consistently exceed the established DRLs, corrective actions, such as adjusting practices or reconfiguring the x-ray systems, are recommended [10,11].

While studies conducted in radiology departments suggest that such audits positively impact imaging practices [12,13,14,15], there is limited data on the efficacy and safety of radiation audits in cardiology departments [9]. The first clinical radiation audit in a cardiology department in Switzerland took place in the electrophysiology unit of the University Hospital Zurich in 2019. We conducted a retrospective cohort study to compare local DRLs for specific cardiac electrophysiology (EP) procedures to the currently available national DRLs and to investigate whether patient radiation exposure was affected by the clinical audit.

We present radiation data from a tertiary care center covering a wide range of EP procedures and, to the best of our knowledge, are one of the first to report on the impact of a clinical radiation audit on patient radiation exposure for specific EP procedures. Our findings aim to enhance safety and improve imaging practices in cardiac electrophysiology.

## Methods

**Study Design and Patient Population** All patients undergoing an electrophysiological procedure between October 2018 and October 2020 were included in our retrospective cohort single center study. The approval of the ethics committee (BASEC-Nr. 2022-01326) was obtained and the study was conducted in accordance with the ethical standards laid down in the Declaration of Helsinki. We followed the STROBE reporting guidelines, with further information found in the online data supplement.

Electrophysiological procedures included pulmonary vein isolation (PVI) with the cryoballoon or with radiofrequency (RF) RF ablation of right-sided supraventricular tachycardia, RF ablation of left-sided supraventricular tachycardia, RF ablation of right-sided ventricular arrhythmias, RF ablation of left-sided ventricular arrhythmias and diagnostic electrophysiology studies (EPS), with or without 3D mapping. We excluded incorrectly included patients (for example twice or cancelled procedure), patients with adult congenital heart disease electrophysiology procedures or not clearly classifiable procedures due to low procedure numbers and widely heterogenous procedures and those with missing data (Supplemental Fig. S1).

### Procedures and Outcomes

**The clinical radiation audit.** In Switzerland, the first clinical radiation audit in a cardiology department took place at the University Hospital Zurich in September 2019. The team of external auditors consisted of a cardiac electrophysiologist, an interventional cardiologist, a medical physicist, and an allied professional working in the catheterization laboratory. A quality manual that described how radiation protection topics are organized including procedures and processes of the department was sent to the auditors before their visit. During the visit the auditor team discussed common matters with the members of the audited department. Additionally, the audit included the observation of procedures. At the end of the audit, each auditor focused on their area of expertise and compared the practices of their peers against recognized standards. Auditor recommendations for clinical practice improvements were provided in a report. Along with the evaluation of patient doses through the use of local DRLs, the audit report suggested the regular use of collimation, semitransparent filter, the reduction of cine images and frame rates, as well as radiation protection recommendations for the personnel.

**Outcomes.** Patient radiation exposure was assessed by the cumulative dose (also known as the incident air kerma at patient reference point (K_a,r_) in mGy), the fluoroscopy time (t in seconds), the dose-area product (DAP in Gy cm^2^) and the effective dose (E in mSv).

K_a,r_ used to estimate the patients’ tissue reactions, is the air-kerma measured at 15 cm from the isocenter towards the x-ray tube. The reference point corresponds to the point where x-ray beams enter a medium-sized patient, in practice the skin of the patient’s back. [9]. The fluoroscopy time corresponds to the time that the x-ray tube was on including the time for fluoroscopy and cine acquisitions [9]. E was calculated with the DAP of each procedure and a conversion coefficient using the ICRP 103 (0.212 mSv/ Gy cm^2^) [10]. E provides an estimation of a whole-body exposure and is mainly used to compare different examinations in terms of dose.

As secondary endpoints, the acute procedural success rate and major 30-day complications were assessed. Major complications were defined as transient ischemic attack, stroke, hemodynamically relevant pericardial effusion, femoral access site complications, acute heart failure, phrenic nerve injury, accidental complete AV block and cardiovascular death occurring within 30 days after the procedure. Electronic health records were reviewed for complications.

### Statistical analysis

Data was collected from a dose management system (DMS, DOSE, Qaelum NV, Belgium) and was cross-checked for accuracy with the patient information system. Continuous variables were expressed as medians and interquartile ranges, categorical variables as counts and percentages. Continuous variables were compared with the Mann-Whitney-U test, categorical variables with the Fisher‘s exact test.

Third quartile values were used as local diagnostic reference levels (DRLs) [16]. For comparison of the local DRLs to the national DRLs, the procedure classification according to the Swiss Federal Ministry of Health (FOPH) was used and is as followed: diagnostic EPS, AVNRT/AVRT ablation, AVNRT/AVRT with 3D electro-anatomical mapping (EAM) and PVI.

To evaluate the effect of the clinical radiation audit on patient radiation exposure for specific procedures a more representative procedure classification was used: PVI cryoablation, PVI RF ablation, RF ablation of right-sided supraventricular tachycardia, RF ablation of left-sided supraventricular tachycardia, RF ablation of right-sided ventricular arrhythmias, RF ablation of left-sided ventricular arrhythmias, and diagnostic EPS. Comparisons of procedure groups before and after the clinical radiation audit were only performed if a sensitivity power analysis indicated that the minimal effect size required to distinguish groups was smaller than 0.6 at a power level of 0.8. Prior to analysis, conditions of normality and homoscedasticity were checked with the Shapiro-Wilk-test and the f-test, respectively. To evaluate the effect of the clinical radiation audit on patient radiation exposure a nonparametric aligned ranks transformation ANOVA was performed. Post-hoc analyses were Mann-Whitney-U tests carried out between groups. P values for post-hoc comparisons were corrected with the Holm-Bonferroni method.

As secondary endpoints, for efficacy, acute procedural success rate, and for safety, 30-day major complications were assessed. The 30-day major complications correspond to the total number of events, with multiple events allowed per patient.

All hypothesis testing was two-tailed, and an alpha-value of 0.05 was considered as the statistically significant threshold. R studio version 4.2.3 was used to perform all statistical analyses.

## Results

### Baseline Characteristics

Patient flow is presented in Supplemental Fig. 1. Baseline characteristics of the 775 included patients are shown in Table 1. 447 patients had their procedure performed before, and 328 patients after the clinical radiation audit. The types of performed procedures are shown in Table 2. There was no significant difference in the use of 3D EAM before and after the audit.

**Table 1:** **Baseline characteristics** shown for all patients and classified whether the procedure was before or after the audit. Continuous variables are presented as medians [1^st^ and 3^rd^ quartile], categorical variables are presented as numbers (percentage). Continuous variables were compared with the Mann-Whitney-U test, and categorical variables with the Fisher‘s exact test.

	**Overall**(n= 775, 100%)	**Before Audit**(n = 447, 57.7 %)	**After Audit**(n = 328, 42.3%)	**p value**
**Age** (years)	61.0 [51.0-70.0]	60.0 [49.0-69.0]	62.0 [53.0-71.0]	0.08
**Sex** (male)	498 (64.3)	278 (62.2)	220 (67.1)	0.18
**BMI** (kg/ m^2^)	26.0 [23.0-29.3]	25.9 [23.0-28.7]	26.0 [23.0-29.8]	0.14
**Medical history**				
Arterial hypertension	341 (44.1)	198 (44.3)	143 (43.6)	0.88
Dyslipidemia	330 (42.6)	158 (35.3)	145 (44.2)	0.01
Diabetes Mellitus	75 (9.7)	42 (9.4)	33 (10.1)	0.8
Smoking	358 (46.2)	201 (44.97)	157 (47.87)	0.47
Coronary artery disease	123 (15.9)	64 (14.3)	59 (17.99)	0.2
Atrial fibrillation / - flutter	469 (60.5)	249 (55.7)	220 (67.1)	0.001
Left ventricular ejection fraction				
≥ 50 %	594 (76.6)	354 (79.2)	240 (73.2)	0.06
41 – 49 %	60 (7.7)	31 (6.9)	29 (8.8)	0.34
≤ 40 %	119 (15.4)	62 (13.9)	57 (17.4)	0.19
**Preprocedural medication**				
Oral anticoagulation	437 (56.4)	225 (50.3)	212 (64.6)	< 0.001
Platelet aggregation inhibition	76 (9.8)	48 (10.7)	28 (8.5)	0.33
Betablockers	442 (57.0)	242 (54.1)	200 (60.98)	0.07
Antiarrhythmic medication	188 (24.3)	110 (24.6)	78 (23.8)	0.8

**Table 2:** **Types of procedures** shown for all patients and classified whether the procedure was before or after the audit, presented as numbers (percentage). Comparison between groups by the Fisher‘s exact test.

	**Overall**(n= 775, 100%)	**Before Audit**(n = 447, 57.7 %)	**After Audit**(n = 328, 42.3%)	**p value**
**Type of procedure**				
PVI Cryoablation	109 (14.1)	57 (12.8)	52 (15.9)	0.25
PVI RF ablation	207 (26.8)	104 (23.3)	103 (31.4)	0.014
RF ablation right-sided supraventricular tachycardia	249 (32.1)	160 (35.8)	89 (27.1)	0.013
RF ablation left-sided supraventricular tachycardia	38 (4.9)	20 (4.5)	18 (5.5)	0.6
RF ablation right-sided ventricular arrhythmias	20 (2.6)	11 (2.5)	9 (2.7)	0.82
RF ablation left-sided ventricular arrhythmias	58 (7.5)	27 (6.0)	31 (9.5)	0.1
Diagnostic EPS	94 (12.1)	68 (15.2)	26 (7.9)	0.002

### Local DRLs and Comparison to National DRLs

Median values and local DRLs for diagnostic EPS, AVNRT/AVRT ablation, AVNRT/AVRT ablation with EAM, and PVI are shown in Table 3. The currently available national DRLs are presented in the same table to aid in comparison.

**Table 3:** Local diagnostic reference levels (DRLs) and comparison to national DRLs shown for the dose-area product (DAP) (Gy cm^2^), the cumulative dose (mGy), and the fluoroscopy time (s) for diagnostic EPS, AVNRT/AVRT ablation, AVNRT/AVRT ablation with electro-anatomical mapping (EAM) and pulmonary vein isolation (PVI), respectively. The first column shows the numbers of procedures (percentage), the second column the local values presented as medians [1^st^ and 3^rd^ quartile]. Median values were used as typical values. Third quartile values were set as local DRLs [16] and are printed in bold. In the third column national DRLs according to the FOPH are shown.

**Type of procedure**	**Overall**(n=557, 100%)	**Local DRLs**	**National DRLs**
		**DAP**	**(Gy cm^2^)**
Diagnostic EPS	72 (12.9)	0.41 [0.19-**0.99**]	20
AVNRT/AVRT ablation	97 (17.4)	0.51 [0.24-**1.00**]	150
AVNRT/AVRT ablation with EAM	74 (13.3)	0.78 [0.29-**2.00**]	30
PVI	314 (56.4)	8.43 [3.09-**16.76**]	NA
		**Cumulative dose**	**(mGy)**
Diagnostic EPS	72 (12.9)	2.54 [0.97-**6.24**]	300
AVNRT/AVRT ablation	97 (17.4)	6.13 [1.72-**7.85**]	2250
AVNRT/AVRT ablation with EAM	74 (13.3)	6.03 [1.91-**17.00**]	623
PVI	314 (56.4)	61.9 [23.0-**127.04**]	NA
		**Fluoroscopy time**	**(s)**
Diagnostic EPS	72 (12.9)	145.5 [64.75-**297.0**]	600
AVNRT/AVRT ablation	97 (17.4)	215.0 [104.5-**311.5**]	1500
AVNRT/AVRT ablation with EAM	74 (13.3)	173.5 [78.5-**336.0**]	540
PVI	314 (56.4)	515.5 [208.8-**955.2**]	2700

NA: not available

The local DRLs for DAP were far below the national DRLs: for diagnostic EPS 0.99 Gy cm^2^ versus 20 Gy cm^2^, for AVNRT/AVRT ablation 1 Gy cm^2^ versus 150 Gy cm^2^, and for AVNRT/AVRT ablation with EAM 2 Gy cm^2^ versus 30 Gy cm^2^. Similarly, the local DRLs for cumulative dose and fluoroscopy time were far below the national DRLs across all procedure groups (Table 3). National DRLs for the DAP and cumulative dose for PVI are not available yet. Also, national Swiss DRLs for other electrophysiological procedures, like ablation of ventricular tachycardia or premature ventricular contractions, are currently not available.

### Patient Radiation Exposure before and after the clinical radiation audit

For comparison of patient radiation exposure before and after the clinical radiation audit, only groups that provided sufficient power in the power calculation were analyzed (Supplemental Table 1-8). Patient radiation data for all other groups that did not meet the power calculation criteria are shown in Supplemental Table 9.

**Fig. 1:**
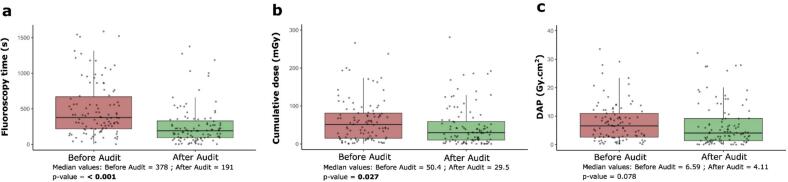
Patient radiation exposure for PVI RF ablation. Box plots showing a the fluoroscopy time (in seconds), b the cumulative dose (in mGy), and c the dose-area product (DAP) (in Gy cm^2^), before and after the clinical radiation audit respectively. Groups were compared with the Mann-Whitney U-Test. P-values were corrected with the Holm-Bonferroni method.

**Fig. 2:**
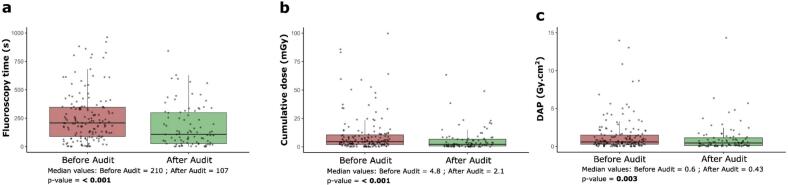
Patient radiation exposure for RF ablation of right-sided supraventricular tachycardia. Box plots showing a the fluoroscopy time (in seconds), b the cumulative dose (in mGy), and c the dose-area product (DAP) (in Gy cm^2^), before and after the clinical radiation audit respectively. Groups were compared with the Mann-Whitney-U test. P-values were corrected with the Holm-Bonferroni method.

The cumulative dose and the fluoroscopy time were significantly reduced for PVI RF ablation after the clinical radiation audit. whereas for DAP there was no significant difference (Fig. 1). For RF ablation of right-sided supraventricular tachycardia there was a significant reduction in patient radiation exposure after the clinical audit, as measured by DAP (0.6 Gy cm^2^ before the audit versus 0.43 Gy cm^2^ after the audit, p-value 0.003), cumulative dose (4.8 mGy before versus 2.09 mGy after, p-value < 0.001) and fluoroscopy time (210 s before versus 107 s after, p-value < 0.001) (Fig. 2).

### Acute procedural success rate and major complications

Overall, acute procedural success rate was 99.2% with no significant differences before and after the clinical radiation audit. Within 30 days after the procedure, in total 28 complications (3.6%) were reported with femoral access site complications being the most common (1.03%), followed by stroke (0.6%) and acute heart failure (0.6%). There was no significant difference in complications before and after the clinical radiation audit, with 3.6% and 3.7 %, respectively (Table 4).

**Table 4:** **Acute procedural success and 30-day major complications** shown for procedures before and after the audit. Data are presented as numbers, in brackets () the percentages. Comparison between groups was made with the Fisher‘s exact test.

	**Overall**(n= 775, 100%)	**Before Audit**(n = 447, 57.7 %)	**After Audit**(n = 328, 42.3%)	**p value**
**Acute procedural success**	769 (99.2)	443 (99.1)	326 (99.4)	1
**Complications**	28 (3.6)	16 (3.6)	12 (3.7)	1
Transient ischemic attack	2 (0.26)	2 (0.4)	0 (0)	
Stroke	5 (0.6)	4 (0.9)	1 (0.3)	
Hemodynamically relevant pericardial effusion	1 (0.13)	0 (0)	1 (0.3)	
Femoral access site complications	8 (1.03)	4 (0.9)	4 (1.2)	
Acute heart failure	5 (0.65)	2 (0.4)	3 (0.9)	
Phenic nerve injury	1 (0.13)	1 (0.2)	0 (0)	
Accidental complete AV block	1 (0.13)	0 (0)	1 (0.3)	
Cardiovascular death	0 (0)	0 (0)	0 (0)	

## Discussion

This retrospective study assessed patient radiation exposure during cardiac electrophysiology procedures by establishing local DRLs and investigating the effect of the first clinical radiation audit performed in a cardiology department in Switzerland. We report **three** major findings:

**First**, our local DRLs were far below the currently available national DRLs (published on the homepage of the Swiss FOPH). DRLs are used as a benchmark to ensure that the amount of ionizing radiation for procedures remains within acceptable levels. For diagnostic electrophysiology studies, our results are consistent with data from existing studies, with the DAP ranging from 0.1 to 3.5 Gy cm^2^ and the fluoroscopy time between 120 to 252 seconds [1,6,7,17]. Similarly, the DAP for PVI, at 8.4 Gy cm^2^, aligns with findings in the literature, which range from 7.1 to 31 Gy cm^2^ [3,6]. For AVNRT/AVRT ablation, we report a DAP of 0.5 - 0.8 Gy cm^2^ and a fluoroscopy time of 173.5 - 215 seconds, which are below the values presented in a large retrospective analysis over a seven-year period [17]. Currently, the FOPH does not provide reference levels for catheter ablation of ventricular tachycardia or premature ventricular contractions. Given the advances in x-ray systems, fluoroscopy protocols, and 3D-mapping systems over recent years, and the associated reduction in radiation [3], our findings highlight the need for regularly updating local and national DRLs.

Additionally, we suggest a more practical approach to classifying procedures for DRLs. Considering that the transseptal puncture (besides the placement of the catheters) is one of the main sources of radiation in many electrophysiology procedures [3], differentiating between right-sided and left-sided EP procedures reasonable.

**Second**, we report a significant reduction of patient radiation exposure after the clinical radiation audit for RF ablation of right-sided supraventricular tachycardia and PVI with RF energy. We attribute this improvement to enhanced operator practices implemented after the audit, such as limiting the use of cine images, considering that the radiation dose in cine mode can be 10 times higher than in fluoroscopic mode [3,4]. Further, we aimed to use collimation consistently and maintain a low frame rate of 3 frames per second. The use of lower frame rates resulted in a reduction in radiation exposure of up to 40% [18], while collimation to the minimum required visual field led to a reduction in radiation dose of up to 37%, as demonstrated in a recent study of 205 EP procedures [19]. Interestingly, while the clinical radiation audit led to a reduction in patient radiation exposure for PVI RF ablations, no significant changes in radiation dose for PVI cryoablation were observed. This may be explained by the observation that our radiation doses for cryoballoon-based PVI before the audit were already relatively low as suggested by the rather low DAP of 8.4 Gy cm^2^ for all PVI procedures as compared to current literature [20], which reduces the scope for further radiation reduction. This is particularly true for cryoballoon-based PVI, which needs fluoroscopy to assess balloon occlusion. Another aspect is that ICE is not routinely used in Switzerland due to its high costs, which may further help to reduce radiation [20].

As demonstrated in a recent study by our group on clinical radiation audits for cardiac implantable electronic devices [9], our findings highlight that audit recommendations effectively address deficiencies in imaging practices and encourage the audited department to continue improving operator radiation practices.

**Third**, there was no significant difference in acute procedural success rate and major complications before and after the clinical radiation audit. The acute procedural success rate, indicating efficacy, remained high at 99.2%. Regarding safety, the 30-day major complication rate was 3.6%, aligning with the expected range of 2.2-10.2% [21,22,23,24,25] with no significant difference observed before and after the audit, which indicates that our measures to reduce radiation exposure were safe.

### Limitations

The study‘s retrospective, single-center design could limit the validity of its findings and the generalizability of the results. Conducted in a tertiary care center, the procedures performed were diverse and varied in complexity, making the establishment of DRLs more challenging but also more reflective of real-word practice. The experience of the operators might be a confounding factor; in our study, all main operators had more than five years of experience in conducting electrophysiology procedures. ICE is not routinely used in our center due to its high costs, which may further help to reduce radiation in cardiac EP procedures. Ultimately, we recognize that a prospective multicenter study would provide more robust, generalizable, and accurate findings.

## Conclusions

The clinical radiation audit in our department was associated with a significant reduction of patient radiation exposure for right-sided supraventricular tachycardia ablation and PVI with RF energy without compromising efficacy and safety. Additionally, we found that our local DRLs were far below national DRLs suggesting that national DRLs need to be updated more regularly to reflect current technological advancements and best practices. Our study highlights the importance of awareness regarding radiation exposure in the EP lab and shows that clinical radiation audits might be an effective tool for optimizing radiation use.

## Consent

This retrospective study was approved by the ethics committee (BASEC-Nr. 2022-01326).

## CRediT authorship contribution statement

**Lorraine Sazgary:** Writing – review & editing, Writing – original draft, Visualization, Methodology, Investigation, Formal analysis, Data curation, Conceptualization. **Eleni Theano Samara:** Writing – review & editing, Methodology, Data curation, Conceptualization. **Anja Stüssi:** Writing – review & editing, Conceptualization. **Natalia Saltybaeva:** Writing – review & editing, Data curation. **Matthias Guckenberger:** Writing – review & editing. **F. Ruschitzka:** Writing – review & editing. **Thomas Wolber:** Writing – review & editing, Data curation. **Nadine Molitor:** Writing – review & editing, Data curation. **Fu Guan:** Writing – review & editing, Data curation. **Gonca Suna:** Writing – review & editing, Data curation. **Julia Hermes-Laufer:** Writing – review & editing, Data curation. **Alexander Breitenstein:** Writing – review & editing, Data curation. **Corinna B. Brunckhorst:** Writing – review & editing, Data curation. **Firat Duru:** Writing – review & editing, Data curation. **Ardan M. Saguner:** Writing – review & editing, Writing – original draft, Visualization, Validation, Supervision, Resources, Project administration, Methodology, Investigation, Data curation, Conceptualization.

## Declaration of Generative AI and AI-assisted technologies in the writing process

During the preparation of this work the author(s) used ChatGPT in order to improve language and readability. After using this tool, the author(s) carefully reviewed and edited the content as needed and take full responsibility for the content of publication.

## Funding

Our study received no funding.

## Declaration of competing interest

The authors declare that they have no known competing financial interests or personal relationships that could have appeared to influence the work reported in this paper.
