# Supplementary material for: Clinical radiation audits as a tool for the optimization of radiation exposure in cardiac electrophysiology procedures
Source: Z Med Phys. 2025 Apr 25;36(2):247–52. doi: 10.1016/j.zemedi.2025.04.003 (PMC13316510; doi:10.1016/j.zemedi.2025.04.003)
Supplement: Supplementary Data 1 [file mmc1.docx]

Supplementary Material to:

**Clinical Radiation Audits as a Tool for the Optimization of Radiation Exposure in Cardiac Electrophysiology Procedures**

Lorraine Sazgary ^1^, Eleni Theano Samara ^2^, Anja Stüssi ^2^, Natalia Saltybaeva ^2^, Matthias Guckenberger ^3^, F Ruschitzka ^1^, Thomas Wolber ^1^, Nadine Molitor ^1^, Fu Guan^1^, Gonca Suna^1^, Julia Hermes-Laufer ^1^, Alexander Breitenstein ^1^, Corinna B. Brunckhorst ^1^, Firat Duru ^1,4,5^, Ardan M. Saguner ^1,4^

^1^ Department of Cardiology, University Hospital Zurich, Zurich, University of Zurich, Switzerland, ^2^ Radiation Protection Unit, University Hospital Zurich, University of Zurich, Zurich, Switzerland, ^3^ Department of Radiation Oncology, University Hospital Zurich, University of Zurich, Zurich, Switzerland, ^4^ Center for Translational and Experimental Cardiology (CTEC), University of Zurich, Zurich, Switzerland, ^5^ Center for Integrative Human Physiology, University of Zurich, Zurich, Switzerland

**Correspondence to:**

Ardan M. Saguner, MD

Senior Consultant, Head of Ventricular Arrhythmia Service

Department of Cardiology, University Hospital Zurich, Rämistrasse 100, 8091 Zurich, Switzerland

Phone Number: + 41 43 253 78 85

E-Mail: ardansaguner@gmail.com

**Supplemental Methods**

**STROBE Statement**—Checklist of items that should be included in reports of ***cohort studies***

|  | Item No | Recommendation | Page |
| --- | --- | --- | --- |
| **Title and abstract** | 1 | (a) Indicate the study’s design with a commonly used term in the title or the abstract | 2 |
|  |  | (b) Provide in the abstract an informative and balanced summary of what was done and what was found | 2 |
| Introduction | | |  |
| Background/rationale | 2 | Explain the scientific background and rationale for the investigation being reported | 4, 5 |
| Objectives | 3 | State specific objectives, including any prespecified hypotheses | 4, 5 |
| Methods | | |  |
| Study design | 4 | Present key elements of study design early in the paper | 6 |
| Setting | 5 | Describe the setting, locations, and relevant dates, including periods of recruitment, exposure, follow-up, and data collection | 6 - 8 |
| Participants | 6 | (a) Give the eligibility criteria, and the sources and methods of selection of participants. Describe methods of follow-up | 6 - 8 |
|  |  | (b) For matched studies, give matching criteria and number of exposed and unexposed | x |
| Variables | 7 | Clearly define all outcomes, exposures, predictors, potential confounders, and effect modifiers. Give diagnostic criteria, if applicable | 7 |
| Data sources/ measurement | 8 * | For each variable of interest, give sources of data and details of methods of assessment (measurement). Describe comparability of assessment methods if there is more than one group | 7 |
| Bias | 9 | Describe any efforts to address potential sources of bias | 8, 9 |
| Study size | 10 | Explain how the study size was arrived at | 6 |
| Quantitative variables | 11 | Explain how quantitative variables were handled in the analyses. If applicable, describe which groupings were chosen and why | 6 9 |
| Statistical methods | 12 | (a) Describe all statistical methods, including those used to control for confounding | 8, 9 |
|  |  | (b) Describe any methods used to examine subgroups and interactions | 8, 9 |
|  |  | (c) Explain how missing data were addressed | 6 |
|  |  | (d) If applicable, explain how loss to follow-up was addressed | 6 |
|  |  | (e) Describe any sensitivity analyses | x |
| Results | | |  |
| Participants | 13 * | (a) Report numbers of individuals at each stage of study—eg numbers potentially eligible, examined for eligibility, confirmed eligible, included in the study, completing follow-up, and analysed | 10 |
|  |  | (b) Give reasons for non-participation at each stage | x |
|  |  | (c) Consider use of a flow diagram | 10 |
| Descriptive data | 14 * | (a) Give characteristics of study participants (eg demographic, clinical, social) and information on exposures and potential confounders | 10 |
|  |  | (b) Indicate number of participants with missing data for each variable of interest | x |
|  |  | (c) Summarise follow-up time (eg, average and total amount) | 8 |
| Outcome data | 15 * | Report numbers of outcome events or summary measures over time | 10-11 |
| Main results | 16 | (a) Give unadjusted estimates and, if applicable, confounder-adjusted estimates and their precision (eg, 95% confidence interval). Make clear which confounders were adjusted for and why they were included | 10-11 |
|  |  | (b) Report category boundaries when continuous variables were categorized | 10-11 |
|  |  | (c) If relevant, consider translating estimates of relative risk into absolute risk for a meaningful time period | x |
| Other analyses | 17 | Report other analyses done—eg analyses of subgroups and interactions, and sensitivity analyses | 10-11 |
| Discussion | | |  |
| Key results | 18 | Summarize key results with reference to study objectives | 12, 14 |
| Limitations | 19 | Discuss limitations of the study, taking into account sources of potential bias or imprecision. Discuss both direction and magnitude of any potential bias | 14 |
| Interpretation | 20 | Give a cautious overall interpretation of results considering objectives, limitations, multiplicity of analyses, results from similar studies, and other relevant evidence | 12-14 |
| Generalizability | 21 | Discuss the generalizability (external validity) of the study results | 12-14 |
| Other information | | |  |
| Funding | 22 | Give the source of funding and the role of the funders for the present study and, if applicable, for the original study on which the present article is based | 15 |

*Give information separately for exposed and unexposed groups.

Source: https://www.strobe-statement.org /

**Definitions**

**Further Baseline Definitions**

**Smoking** was defined as current smoking or smoking in the past.

**Coronary artery disease** was defined as history of coronary artery disease, history of acute myocardial infarction, or finding of ≥ 50% stenosis on coronary angiogram.

**Atrial fibrillation/ - flutter** was defined as a history of symptomatic or asymptomatic atrial fibrillation / -flutter lasting ≥30s documented by ECG.

**Left ventricular ejection fraction** was classified in ≥ 50 %, 41 – 49 %, ≤ 40 %. Echocardiographic measurements of left ventricular systolic function were performed according to the current echocardiographic guidelines on chamber quantification for adults ^1^.

**Radiofrequency (RF) ablation right-sided supraventricular tachycardia** included AVNRT ablation, ablation of focal/multifocal right-atrial tachycardia, ablation of cavotricuspid isthmus-dependent macro-re-entrant atrial tachycardia, ablation of non-cavotricuspid isthmus-dependent macro-re-entrant right-atrial tachycardia, ablation of right-sided accessory pathways in AVRT, and AV node ablations.

**RF ablation left-sided supraventricular tachycardia** included ablation of focal/multifocal left-atrial tachycardia, ablation of non-cavotricuspid isthmus-dependent macro-re-entrant left-atrial tachycardia, and ablation of left-sided accessory pathways in AVRT.

**RF ablation right-sided ventricular arrhythmias** included ablation of ventricular tachycardia and premature ventricular contractions originating from the right ventricle.

**RF ablation left-sided ventricular arrhythmias** included ablation of ventricular tachycardia and premature ventricular contractions originating from the left ventricle.

**Further Outcome Definitions**

**Cardiovascular death** includes death due to acute myocardial infarction, sudden cardiac death, heart failure, stroke, cardiovascular procedure, cardiovascular hemorrhage (for example aortic aneurysm or dissection), and pulmonary embolism.

**Hemodynamically relevant pericardial effusion** was defined according to clinical and echocardiographic findings. As echocardiographic criteria, those recommended by the current guidelines ^2^, were used, including the collapsing of cardiac chambers, a dilated (≥ 2.1 cm) Vena cava inferior with < 50% reduction in diameter during inspiration, respirophasic abnormal ventricular septum motion, and increased respiratory variation in transvalvular velocities (for peak mitral E inflow exceeding > 30% respiratory variation; for peak tricuspid E inflow exceeding > 60% respiratory variation).

**Acute heart failure** was defined by typical symptoms (such as edema or dyspnea) and signs (such as positive hepatojugular reflux) and elevated nt-pro BNP levels ^3^. When requiring admission to the hospital or causing a prolongation of hospitalization, it was defined as major complication.

**Femoral access site complications** included hematoma, pseudo aneurysm and arteriovenous fistula. They were considered as major if they led to extended hospitalization, re-hospitalization, blood transfusion, percutaneous thrombin injection, or surgical intervention.

**X-ray system**

All procedures were performed with a bi-plane cardiology system Artis Q.zen biplane (Siemens Healthcare AG) equipped with two flat-panel detectors 20x20 cm.

**3D-mapping system**

For electro-anatomical mapping the CARTO system (Biosense Webster) was used.

**Supplemental Figures**

**Supplemental Figure 1. Patient flow chart** shown for the 850 patients included. The reasons for exclusion and the absolute numbers of patients excluded are shown in the boxes on the right. 775 patients were included in the final analyses.


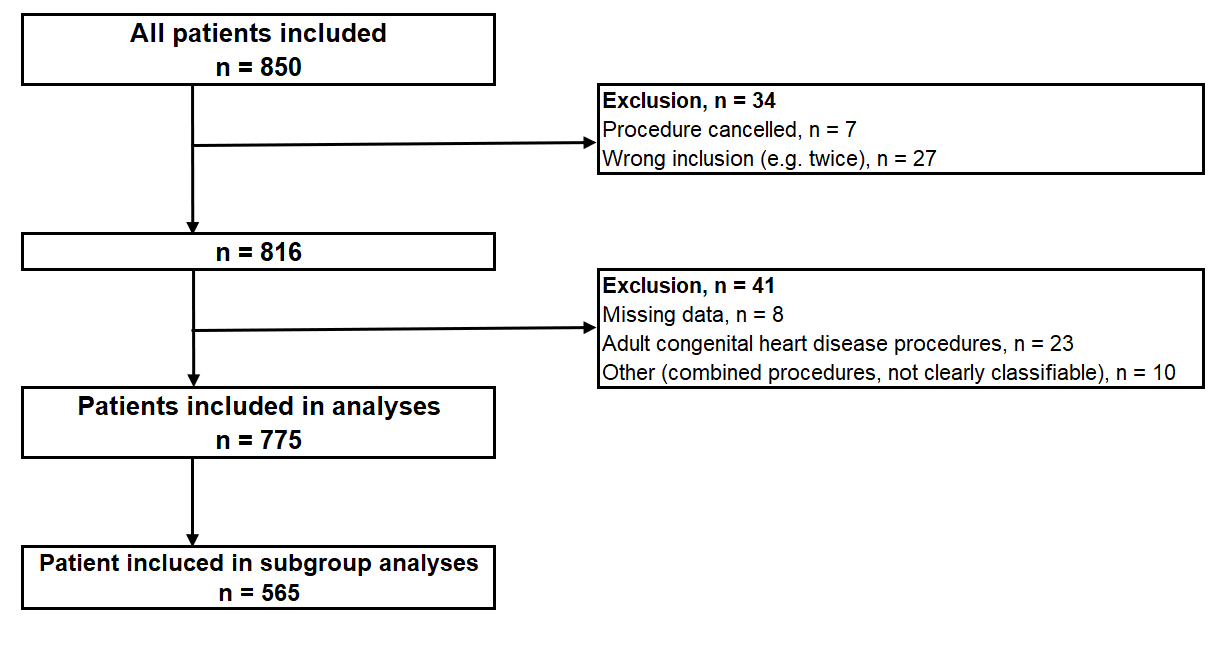


**Supplemental Tables**

1. The effect of the clinical radiation audit on **dose-area product** (DAP)**.**

**Supplemental Table 1.** Nonparametric two-way ANOVA test on aligned rank transformed data following this formula: DAP ~ label*procedure. Label was defined as before and after the clinical radiation audit.

**Dose-area product (Gy cm^2^)**

| **Term** | **Df** | **Df.res** | **F value** | **p value** |
| --- | --- | --- | --- | --- |
| Label | 1 | 559 | 16.27 | < 0.001 |
| Procedure | 2 | 559 | 296.26 | < 0.001 |
| Label:procedure | 2 | 559 | 11.13 | < 0.001 |

**Supplemental Table 2** showing DAP for the different types of procedures before and after the clinical radiation audit. Date are presented as medians, and 1^st^ and 3^rd^ quartile in brackets []. Outliers above the three standard deviation interval were removed. Groups were compared with the Mann-Whitney-U test. P-values were corrected with the Holm-Bonferroni method.

**Dose-area product (Gy cm^2^)**

| **Type of procedure** | **Overall**  ( n= 565, 100%) | **Before Audit**  (n = 321, 56.8 %) | **After Audit**  (n = 244, 43.2%) | **p-value** |
| --- | --- | --- | --- | --- |
| PVI Cryoablation | 17.55 [9.91 -28.56] | 14.43 [8.73 – 25.2] | 20.04 [12.8 – 33.35] | 0.078 |
| PVI RF ablation | 4.95 [1.81 – 9.77] | 6.59 [2.63 – 11.0] | 4.11 [1.35 – 9.17] | 0.078 |
| RF ablation right-sided supraventricular tachycardia | 0.54 [0.21 – 1.35] | 0.6 [0.28 – 1.55] | 0.43 [0.11 – 1.04] | **0.003** |

1. The effect of the clinical radiation audit on **cumulative dose**.

**Supplemental Table 3** Nonparametric two-way ANOVA test on aligned rank transformed data following this formula: Cumulative dose ~ label*procedure. Label was defined as before and after the clinical radiation audit.

**Cumulative dose (mGy)**

| **Term** | **Df** | **Df.res** | **F value** | **p value** |
| --- | --- | --- | --- | --- |
| Label | 1 | 559 | 9.34 | 0.002 |
| Procedure | 2 | 559 | 298.5 | < 0.001 |
| Label:procedure | 2 | 559 | 7.69 | < 0.001 |

**Supplemental Table 4** showing the cumulative dose for the different types of procedures before and after the clinical radiation audit. Date are presented as medians, and 1^st^ and 3^rd^ quartile in brackets []. Outliers above the three standard deviation interval were removed. Groups were compared with the Mann-Whitney-U test. P-values were corrected with the Holm-Bonferroni method.

**Cumulative dose (mGy)**

| **Type of procedure** | **Overall**  ( n= 565, 100%) | **Before Audit**  (n = 321, 56.8 %) | **After Audit**  (n = 244, 43.2%) | **p-value** |
| --- | --- | --- | --- | --- |
| PVI Cryoablation | 147.08 [82.02 - 255.74] | 131.65 [76.67 -222.81] | 156.89 [111.05 – 271.32] | 0.18 |
| PVI RF ablation | 38.65 [12.05 -76.16] | 50.36 [14.9 – 97.99] | 29.5 [10.17 – 59.15] | **0.027** |
| RF ablation right-sided supraventricular tachycardia | 3.74 [1.19 – 8.81] | 4.79 [1.93 – 10.81] | 2.09 [0.68 – 6.11] | **<0.001** |

1. The effect of the clinical radiation audit on **fluoroscopy time**.

**Supplemental Table 5** Nonparametric two-way ANOVA test on aligned rank transformed data following this formula: Fluoroscopy time ~ label*procedure. Label was defined as before and after the clinical radiation audit.

**Fluoroscopy time (s)**

| **Term** | **Df** | **Df.res** | **F value** | **p value** |
| --- | --- | --- | --- | --- |
| Label | 1 | 559 | 17.8 | < 0.001 |
| Procedure | 2 | 559 | 173.89 | < 0.001 |
| Label:procedure | 2 | 559 | 3.52 | 0.03 |

**Supplemental Table 6** showing the fluoroscopy time for the different types of procedures before and after the clinical radiation audit. Date are presented as medians, and 1^st^ and 3^rd^ quartile in brackets []. Outliers above the three standard deviation interval were removed. Groups were compared with the Mann-Whitney-U test. P-values were corrected with the Holm-Bonferroni method.

**Fluoroscopy time (s)**

| **Type of procedure** | **Overall**  ( n= 565, 100%) | **Before Audit**  (n = 321, 56.8 %) | **After Audit**  (n = 244, 43.2%) | **p-value** |
| --- | --- | --- | --- | --- |
| PVI Cryoablation | 956 [758 -1364] | 1066 [794.2 – 1506.5] | 870.5 [732 – 1141.5] | **0.037** |
| PVI RF ablation | 268.5 [139.2 -506.2] | 378 [219 – 683.5] | 191 [92.5 – 330.5] | **<0.001** |
| RF ablation right-sided supraventricular tachycardia | 178 [67.5 – 227.1] | 210 [90 – 349.2] | 107 [26.5 – 287.5] | **< 0.001** |

1. The effect of the clinical radiation audit on **effective dose**.

**Supplemental Table 7.** Nonparametric two-way ANOVA test on aligned rank transformed data following this formula: Effective dose ~ label*procedure. Label was defined as before and after the clinical radiation audit.

**Effective dose (mSv)**

| **Term** | **Df** | **Df.res** | **F value** | **p value** |
| --- | --- | --- | --- | --- |
| Label | 1 | 559 | 17.77 | < 0.001 |
| Procedure | 2 | 559 | 313.34 | < 0.001 |
| Label:procedure | 2 | 559 | 11.76 | < 0.001 |

**Supplemental Table 8** showing the effective dose for the different types of procedures before and after the clinical radiation audit. Date are presented as medians, and 1^st^ and 3^rd^ quartile in brackets []. Outliers above the three standard deviation interval were removed. Groups were compared with the Mann-Whitney-U test. P-values were corrected with the Holm-Bonferroni method.

**Effective dose (mSv)**

| **Type of procedure** | **Overall**  ( n= 565, 100%) | **Before Audit**  (n = 321, 56.8 %) | **After Audit**  (n = 244, 43.2%) | **p-value** |
| --- | --- | --- | --- | --- |
| PVI Cryoablation | 3.72 [2.1 – 6.05] | 3.06 [1.85 – 5.35] | 4.25 [2.72 – 7.07] | 0.08 |
| PVI RF ablation | 1.07 [0.39 – 2.19] | 1.40 [0.56 – 2.33] | 0.87 [0.29 – 1.94] | 0.08 |
| RF ablation right-sided supraventricular tachycardia | 0.11 [0.04 – 0.27] | 0.13 [0.06 – 0.30] | 0.09 [0.02 – 0.22] | 0.054 |

**Supplemental Table 9.** DAP, cumulative dose, fluoroscopy time and effective dose shown for RF ablation left-sided supraventricular tachycardia, RF ablation right-sided ventricular arrythmias, RF ablation left-sided ventricular arrhythmias and diagnostic EPS before and after the clinical radiation audit, respectively.

| **Type of procedure** | **Overall**  (n= 210, 100%) | **Before Audit**  (n = 126, 60 %) | **After Audit**  (n = 84, 40%) |
| --- | --- | --- | --- |
|  |  | **DAP (Gy cm^2^)** |  |
| RF ablation left-sided supraventricular tachycardia | 1.51 [0.69 – 2.89] | 1.43 [0.55 – 3.11] | 1.92 [1.05 – 2.66] |
| RF ablation right-sided ventricular arrhythmias | 0.96 [0.07 – 1.61] | 1.13 [0.17 – 1.61] | 0.96 [0.07 – 1.69] |
| RF ablation left-sided ventricular arrhythmias | 9.36 [2.37 – 16.25] | 8.37 [3.28 – 14.95] | 9.36 [1.28 – 20.78] |
| Diagnostic EPS | 0.42 [0.21 – 1.17] | 0.42 [0.18 – 1.17] | 0.37 [0.22 – 1.17] |
|  |  | **Cumulative dose (mGy)** |  |
| RF ablation left-sided supraventricular tachycardia | 14.63 [5.43 – 18.17] | 13.19 [4.12 – 18.22] | 16.36 [8.44 – 18.10] |
| RF ablation right-sided ventricular arrhythmias | 6.51 [0.33 – 10.02] | 7.75 [0.85 -10.21] | 6.51 [0.37 – 10.22] |
| RF ablation left-sided ventricular arrhythmias | 61.26 [17.23 -101.01] | 63.05 [27.61 – 97.53] | 58.70 [9.47 -117.3] |
| Diagnostic EPS | 2.66 [1.15 – 7.43] | 2.69 [1.15 – 6.91] | 2.10 [1.24 – 8.68] |
|  |  | **Fluoroscopy time (s)** |  |
| RF ablation left-sided supraventricular tachycardia | 145.5 [61.3 – 336.3] | 191 [120.2 - 363] | 104 [54.5 - 237] |
| RF ablation right-sided ventricular arrhythmias | 391 [43 – 609.8] | 513 [201.5 – 629.5] | 55 [44 - 549] |
| RF ablation left-sided ventricular arrhythmias | 1020 [383 – 1391.2] | 1038 [348 – 1355.5] | 1002 [385 – 1458.5] |
| Diagnostic EPS | 180.5 [74.75 -358.5] | 172 [74.2 – 328.5] | 184 [85.75 – 361.5] |
|  |  | **Effective dose (mSv)** |  |
| RF ablation left-sided supraventricular tachycardia | 0.32 [0.15 – 0.61] | 0.30 [0.14 – 0.66] | 0.41 [0.22 – 0.56] |
| RF ablation right-sided ventricular arrhythmias | 0.20 [0.01 – 0.34] | 0.24 [0.04 – 0.34] | 0.20 [0.02 – 0.36] |
| RF ablation left-sided ventricular arrhythmias | 1.98 [0.50 – 3.45] | 1.77 [0.69 – 3.17] | 1.98 [0.31 – 4.40] |
| Diagnostic EPS | 0.09 [0.05 – 0.25] | 0.09 [0.04 – 0.25] | 0.08 [0.05 – 0.25] |

**Supplemental References**

1. Lang RM, Badano LP, Mor-Avi V, et al. Recommendations for Cardiac Chamber Quantification by Echocardiography in Adults: An Update from the American Society of Echocardiography and the European Association of Cardiovascular Imaging. *J Am Soc Echocardiogr*. 2015;28(1):1-39.e14. doi:10.1016/j.echo.2014.10.003

2. Al K, S A, Da A, et al. American Society of Echocardiography clinical recommendations for multimodality cardiovascular imaging of patients with pericardial disease: endorsed by the Society for Cardiovascular Magnetic Resonance and Society of Cardiovascular Computed Tomography. *J Am Soc Echocardiogr Off Publ Am Soc Echocardiogr*. 2013;26(9). doi:10.1016/j.echo.2013.06.023

3. McDonagh TA, Metra M, Adamo M, et al. 2021 ESC Guidelines for the diagnosis and treatment of acute and chronic heart failure. *Eur Heart J*. 2021;42(36):3599-3726. doi:10.1093/eurheartj/ehab368
